# Does gallbladder angle affect gallstone formation?

**DOI:** 10.11604/pamj.2016.24.165.7768

**Published:** 2016-06-27

**Authors:** Bekir Sanal, Mehmet Korkmaz, Sezgin Zeren, Fatma Can, Ferhan Elmali, Zulfu Bayhan

**Affiliations:** 1 Department of Radiology, Dumlupinar University Faculty of Medicine, 43100, Kutahya, Turkey; 2 Department of General Surgery, Dumlupinar University Faculty of Medicine, 43100, Kutahya, Turkey; 3 Department of Biostatistics and Bioinformatics, Erciyes University Faculty of Medicine, 38100, Kayseri, Turkey

**Keywords:** Gallstone, gallbladder angle, computed tomography, ultrasonography

## Abstract

**Introduction:**

Morphology of gallbladder varies considerably from person to person. We believe that one of the morphological variations of gallbladder is the “gallbladder angle”. Gallbladder varies also in “angle”, which, to the best of our knowledge, has never been investigated before. The purpose of this study was to investigate the impact of gallbladder angle on gallstone formation.

**Methods:**

in this study, 1075 abdominal computed tomography (CT) images were retrospectively examined. Patients with completely normal gallbladders were selected. Among these patients, those with both abdominal ultrasound and blood tests were identified in the hospital records and included in the study. Based on the findings of the ultrasound scans, patients were divided into two groups as patients with gallstones and patients without gallstones. Following the measurement of gallbladder angles on the CT images, the groups were statistically evaluated.

**Results:**

The gallbladder angle was smaller in patients with gallstones (49 ± 21 degrees and 53 ± 19 degrees) and the gallbladder with larger angle was 1.015 (1/0.985) times lower the risk of gallstone formation. However, these were not statistically significant (p>0,05).

**Conclusion:**

A more vertically positioned gallbladder does not affect gallstone formation. However, a smaller gallbladder angle may facilitate gallstone formation in patients with the risk factors. Gallstones perhaps more easily and earlier develop in gallbladders with a smaller angle.

## Introduction

**Figure 1:**
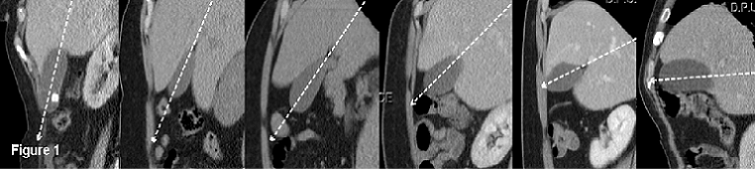
Gallbladder angle varies considerably from person to person on sagittal reformatted CT images

Gallbladder contraction is induced by cholecystokinin that is secreted principally in the small intestine. Cholecystokinin secretion is mainly stimulated by fatty food that is taken in orally. Gallbladder contraction induced by cholecystokinin secretion is followed by the emptying of the stored bile. Impairment or failure of this mechanism causes gallbladder hypomotility [1, 2]. In addition to many risk factors which primarily cause increased cholesterol saturation in bile such as genetic factors, obesity, sudden weight loss, hyperlipidemia and high calorie diet, gallbladder hypomotility, followed by delayed gallbladder emptying and biliary stasis, plays a significant role in gallstone formation [3–6]. It is known that gallbladder emptying is delayed in the luteal phase of menstruation, pregnancy and perimenopausal period. In addition, the incidence of gallstones is particularly increasing among women receiving hormone replacement therapy with progesterone due to delayed gallbladder emptying [5, 7]. It has been shown that gallbladder residual volume is increased and gallbladder emptying isdecreased in diabetic patients [8]. Furthermore, diseases such as chronic liver disease and beta-thalassemia major and surgeries such as gastrectomy and vagotomy are known to produce a direct effect on contractility of gallbladder, leading to gallstone formation [9]. In addition to the above mentioned conditions affecting the gallbladder motility and leading to gallstone formation, such gallbladder anomalies as left-sided, retrohepatic, suprahepatic, intrahepatic and floating gallbladders and gallbladders with real septa have been reported to increase the incidence of gallstone formation by causing biliary stasis without impairing the gallbladder mobility [10–12]. These gallbladder anomalies are quite rare (0,026 %); however, it is quite common that gallbladder varies in size, shape and axis in healthy people [10]. Gallbladder varies also in ‘angle’, which, to the best of our knowledge, has never been investigated before. On sagittal reformatted computed tomography (CT) images, a majority of gallbladders are positioned somewhere in 90-degree-angle between anterior and inferior points. This angle, which is called gallbladder angle, varies considerably from person to person (Figure 1). It is known that stasis of a fluid in the body due to gravity predisposes the stone disease, as in urinary system [13] and submandibular duct [14]. Given that a healthy person spends a significant part of the day on foot or sitting, it can be assumed that biliary passages slows down due to gravity in a more vertically positioned gallbladder, resulting in partial biliary stasis and predisposition to gallstones. In this respect, the purpose of this study was to investigate the impact of gallbladder angle on gallstone formation by comparing patients with gallstones to control subjects without gallstones both of whom had completely normal gallbladders.

## Methods

**Figure 2:**
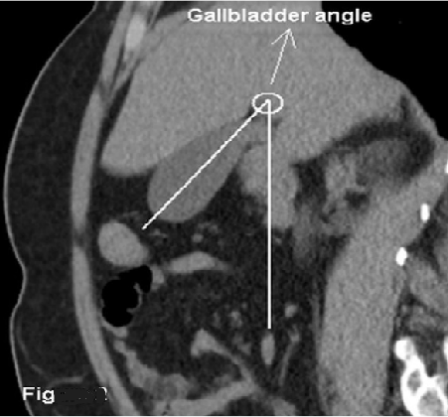
Gallbladder angle is the angle between the line which longitudinally halves the gallbladder from the fundus and the patient table on the clearest longitudinal image of the gallbladder in the sagittal plane

A total of 1075 abdominal CT images (Toshiba Alexion Advance, 16 slice, TSX-034A/1C, JAPAN) which were taken by the Radiology Department of our Hospital for any indication between 01.10.2013 – 01.03.2014 were retrospectively examined. Images belonging to patients over the age of 18 were selected for the study. Preliminary diagnoses of the patients were mainly as follows: urinary stone, acute appendicitis, trauma, malignancy, characterization of a lesion detected by ultrasonography, acute pancreatitis and nonspecific abdominal pain. Some of the CT scans were performed without contrast whereas some were performed with IV contrast only and the rest were performed with a combination of IV, oral and rectal contrast. Based on the reformatted images in all three planes, patients with gross abdominal pathologies, particularly acute cholecystitis, edema/inflammation of gallbladder, liver cirrhosis and liver masses and patients with abdominal scars which might represent intraabdominal surgeries were identified and excluded from the study. Later on, gallbladder morphology and axes were examined, and gallbladders extending in parallel to the section plane were identified on the multiplanar reformatted images in the sagittal plane. Gallbladders oriented to the right and left and extending at parasagittal directions, twisted gallbladders, folded gallbladders, contracted and inflamed gallbladders and gallbladders pushed or displaced by the liver or the other neighboring anatomical structures were excluded from the study. The remaining 378 patients were listed. Hospital records of these 378 patients were investigated in an effort to identify those with abdominal ultrasound scans, hemograme and biochemistry reports. As a result, it was found that 210 patients had an ultrasound scan between 01 01 2013 and 30 09 2014. Of these 210 patients, 165 had also blood tests as well as CT scans performed between the above mentioned dates. Patients other than these 165 patients were excluded from the study. Of these 165 patients with CT scans, blood tests and ultrasound scans dated between 01 01 2013 and 30 09 2014, 15 more were excluded from the study (7 with elevated liver function test values, 4 with elevated inflammatory markers and 4 with anemia). Ultrasound scans (Logiq 7, GE 76 Medical System, Milwaukee, WI, USA) of the remaining 150 patients were examined, and it was found that 52 had a pathological gallbladder whereas 98 had a normal gallbladder. Of these 52 patients, 11 had condensed bile, biliary sludge, cholesterol crystals, gallbladder polyps or acute cholecystitis. Of the remaining 41 patients, 40 had gallstones that met the standard criteria and 1 had a history of cholecystectomy due to gallstones. These 41 patients had a normal gallbladder volume, wall thickness and pericholecystic region, and none of them had biliary dilatation. Besides, they had no reported accompanying hepatobiliary pathologies other than diffuse or focal steatosis, simple cysts, typical hemangioma and non-specific calcification in the liver paranchyma. These 41 patients constituted the patient group in this study. On the other hand, 88 patients with a completely normal gallbladder who matched the patient group with regard to age, gender, lipid profile, degree of hepatic steatosis and presence of diabetes constituted the control group. As a result, 129 patients were included in this study. The patients and the control subjects were residing in the same city and were of the same ethnic origin. The personal information of the patients were not shared in this retrospective analysis. Measurements were made on the magnified reformatted CT images in the sagittal plane. Patient table was taken as the reference line. On the clearest longitudinal image of the gallbladder in the sagittal plane, the angle between the line which longitudinally halved the gallbladder from the fundus and the patient table was accepted as the “gallbladder angle” (Figure 1, Figure 2). Some of the patients did not have body mass index (BMI) data in the hospital records. Therefore, subcutaneous fat thickness was measured in 6 points in the transverse CT section passing through the level of umbilicus, namely both paraumbilical regions, corresponding dorsal parasagittal regions and right and left lateral regions, and the average value was calculated. This average value was accepted as subcutaneous abdominal fat thickness, which represented the BMI [15, 16]. Measurements were made in the same medical system (Siso Viewer - V2.9) by consensus between two radiologists with more than 10 years of abdominal CT experience who did not know whether the subjects were in the patient group or the control group. Five patients had radiopaque stones on CT. Data were analyzed in IBM SPSS Statistics 22.0 statistical package. Summary statistics were expressed as (n) for number of units, (%) for percentage, (χ±sd) for mean standard deviation and (M(Q1-Q3)) for median 25^th^ and 75^th^ percentile values. Whether numeric variables demonstrated a normal distribution or not was determined by Shapiro-Wilk test. Comparison of numeric variables between the two groups was made by means of independent samples *t* test in variables which demonstrated a normal distribution and by means of Whitney U test in variables which did not demonstrate a normal distribution. Exact method of the chi-square test was used for comparison of categorical variables. Binary logistic regression analysis was used for determination of factors that had an impact on gallstone formation. A p value smaller than 0.05 was considered statically significant.

## Results

129 patients were included in this study, 58(45%) were males and 71(55%) were females. The mean patient age was 53 ± 9 years. The mean gallbladder angle was 51 ± 20 degrees. There was no statistically significant difference between the patients and the control subjects in age, gender, degree of hepatic steatosis, subcutaneous abdominal fat thickness, lipid profile and presence of diabetes (Table 1). The mean gallbladder angle was 49 ± 21 degrees in the group with gallstones and 53 ± 19 degrees in the group without gallstones. The mean gallbladder angle was smaller in the group with gallstones compared to the group without gallstones; however, the difference between the groups was not statistically significant (p=0,256). Effects of the variables on gallstone formation were investigated by the binary logistic regression analysis (Table 2). It was found by means of the “Enter” method that the larger the gallbladder angle was 1.015 (1/0.985) times lower the risk of gallstone formation was; however, this value was not statistically significant (p=0.145). In addition, no significant variable was left in the model as a result of backward logistic regression analysis.

**Table 1:** Demographic data of the control and gallstone groups

	Groups		*p*
	Gallstone group *n*=41	Control group *n*=88	
Meanage (years)	54.21±14.88	53.50±14.39	0.794
Sex			
Male	19 (46.3)	39 (44.3)	0.830
Female	22 (53.7)	49 (55.7)	
Hepatic steatosis(grade)	1.0 (0.0-1.5)	0.0 (0.0-2.0)	0.362
Subcutaneous abdominal fat thickness (cm)	2.39±0.81	2.37±0.59	0.900
TRG (mg/dL)	188 (133-251.5)	154 ( 104.3-232)	0.194
HDL (mg/dL)	43 (39-50)	46.5 (38-52.8)	0.843
LDL (mg/dL)	118 (99.5-134)	117 (92-143.5)	0.634
Diabetes mellitus			
(+)	29 (70.7)	66 (75.0)	0.608
(-)	12 (29.3)	22 (25.0)	

Abbreviations: TRG; triglyceride HDL; highdensity lipoprotein, LDL; lowdensity lipoprotein

**Table 2:** Enter method of binary logistic regression analysis was used for determination of factors that had an impact on gallstone formation

	Odds	95.0% CI for Odds	*p*
Gallbladder angles	0.985	(0.965-1.005)	0.145
Age (years)	1.001	(0.972-1.031)	0.952
Sex			0.564
Female	1		
Male	1.293	(0.540-3.095)	
Hepatic steatosis(grade)	1.152	(0.717-1.851)	0.559
Subcutaneous abdominal			
fat thickness (cm)	0.989	(0.515-1.900)	0.974
TRG (mg/dL)	1.000	(0.966-1.004)	0.966
HDL (mg/dL)	1.005	(0.971-1.040)	0.776
LDL (mg/dL)	1.004	(0.993-1.016)	0.476
Diabetes mellitus			
(+)	1	(0.515- 3.421)	0.557
(-)	1.328		

## Discussion

The purpose of this study was to find out whether or not a more vertically positioned gallbladder was more prone to biliary stasis and gallstone formation under the influence of gravity. In this respect, “gallbladder angle” between the gallbladder axis and patient vertical axis was investigated, and this angle was found to be smaller in patients with gallstones compared to control subjects without gallstones. In addition, the larger the gallbladder angle was the lower the risk of gallstone formation was. However, these data were not statistically significant. Based on these findings, we considered that more vertically positioned gallbladders might be slightly more prone to gallstones. To best of our knowledge, no such data is available about this issue in the literature so far. Shape, diameter and position of gallbladder fundus and corpus, which stick to the posterior caudal surface of the liver and extend anteriorly and inferiorly between the right and left lobes, may vary considerably from person to person. The known positional differences of gallbladder might be primarily linked to congenital anomalies; might be just variations; or might be secondary to biliary tract malignancies or liver cirrhosis [10]. All the gallbladders included in this study were positioned differently. To the best of our knowledge, clinical significance of the “gallbladder angle” that was described in this study as an anatomical fact has not been investigated in the literature before. Biliary stasis and nucleation of cholesterol crystals occurring as a result of high levels of biliary cholesterol, low levels of bile salts and reduced gallbladder contractility are the main factors affecting gallstone formation [3, 4, 17]. Occurrence of biliary stasis, followed by gallstone formation, is easier in gallbladders with a larger fasting and post-prandial volume [18].

Indeed, dependent fluids inside the body with no flow or insufficient flow are always prone to condensation and sludge. Formation of bladder stones in case of bladder outlet obstruction [19], higher frequency of stones in the lower pole calyx of the kidney under the influence of gravity compared to other calices [20], higher frequency of stones in the submandibular duct due to its dependent position compared to the other salivary glands [14] can be given as examples for the abovementioned suggestion. In this respect, we considered that partial stasis in the bottom point of the fundus might have contributed to gallstone formation in gallbladders with stones which were more vertically positioned compared to gallbladders without stones. Several studies have shown that various diseases and habits affect the gallbladder via different mechanisms, predisposing it to stones. For example, gallbladder may be predisposed to stones in cholecystokinin deficiency in patients receiving long term parenteral nutrition treatment [1], as well as autonomic nervous system dysfunctions in such connective tissue diseases as rheumatoid arthritis and systemic lupus erythematosus [4] and reduced post-prandial gallbladder volume in case of low physical activity and chronic smoking [21, 22]. Irrespective of the primary cause, the main problem in all these examples is the biliary stasis. Although vertical position per se does not affect gallstone formation, it can contribute to a potential stasis in patients with more vertically positioned gallbladders. It has been shown that gallbladder hypomotility has an effect on not only primary stone formation but also stone recurrence after lithotripsy [17, 23].

Berr et al. studied patients with and without recurrent gallstones after lithotripsy that were well-matched in gallbladder composition and found that stones recurred in gallbladders with higher residual volume and lower ejection fraction. Based on their findings, they concluded that the most important factor in stone formation after lithotripsy was insufficient gallbladder emptying [24]. Investigation of gallbladder angle in patients that are monitored for stone recurrence after lithotripsy might provide clear insight into the impact of gallbladder angle on stone formation. This study has some limitations. Firstly, there were only a limited number of patients in the patient group. In this respect, increasing the number of patients with gallstones might contribute to the statistical strength of this study as well as providing data which might be supportive of our hypothesis. Secondly, no chemical analyses could be performed in the gallstones. Thirdly, we did not have patient’s genetic characteristics and medication history, which might have affected the gallbladder motility. Fourthly, this was a retrospective study. Further prospective studies are needed to shed light on the relationship between gallbladder angle and such parameters as fasting gallbladder volume, post-prandial gallbladder volume and ejection fraction.

## Conclusion

As a conclusion, in this study a more vertically positioned gallbladder does not directly affect gallstone formation. Gallbladder works effectively despite the gravity. However, although it is not influential per se, a smaller gallbladder angle might facilitate stone formation in patients with risk factors. It may be considered that gallstones develop perhaps more easily and early in gallbladders with a smaller angle. However, effects of gallbladder angle on biliary stasis and stone formation should be confirmed by further prospective studies with lager case series.

### What is known about this topic

- Morphology of gallbladder varies considerably from person to person;
- It is quite common that gallbladder varies in size, shape and axis in healthy people;
- Gallbladder anomalies have been reported to increase the incidence of gallstone formation by causing biliary stasis without impairing the gallbladder mobility.

### What this study adds

- This study is the first in the literature to investigate the impact of gallbladder angle on gallstone formation;
- Smaller gallbladder angle might facilitate gallstone formation in patients with risk factors;
- Vertically positioned gallbladder does not directly affect gallstone formation.
